# The Twin‐Rooted Anomaly: Endodontic Management of a Rare Case of a Maxillary Central Incisor With Two Roots

**DOI:** 10.1002/ccr3.71779

**Published:** 2025-12-30

**Authors:** Mohsen Aminsobhani, Maryam Babaahmadi

**Affiliations:** ^1^ Department of Endodontics Dental Research Center, AJA Tehran Iran; ^2^ Tehran University of Medical Sciences Tehran Iran; ^3^ Department of Endodontics, School of Dentistry Tehran University of Medical Sciences Tehran Iran

**Keywords:** anatomic variation, case reports, incisor, maxilla, root canal therapy

## Abstract

This case report describes the endodontic management of a maxillary central incisor with two roots, representing a rare anatomical variation. A 73‐year‐old female patient presented with a fractured tooth #21 with a carious crown. Pulp sensibility testing using cold spray elicited a sharp and prolonged painful response, consistent with a diagnosis of symptomatic irreversible pulpitis with normal apical tissues. Conventional periapical radiography suggested an unusual root morphology, which was further evaluated using cone‐beam computed tomography (CBCT). CBCT imaging revealed a bifurcated root configuration with two distinct root canals and separate apical foramina. Root canal treatment was performed under dental operating microscope magnification using a crown‐down preparation technique, followed by obturation with cold lateral compaction. Clinical and radiographic evaluation at the 1‐year follow‐up demonstrated a successful outcome. This case underscores the importance of careful diagnostic assessment and the judicious use of advanced imaging modalities for the detection and management of rare anatomical variations, even in teeth that typically exhibit simple root canal anatomy.

**Key Clinical Message**

Although maxillary central incisors are typically single‐rooted, rare anatomical variations such as bifurcated roots may occur without evident crown anomalies. Thorough diagnostic evaluation, including the selective use of CBCT, is essential for accurate diagnosis and successful endodontic management in such cases.

## 1 Introduction

Endodontic treatment aims to eliminate infection, relieve pain, and preserve natural teeth. However, the root canal system's anatomical complexity may lead to residual infection or inadequate irrigant removal if variations are overlooked. A successful long‐term prognosis requires thorough cleaning and shaping, and three‐dimensional obturation of the root canal system to ensure bacterial eradication and a hermetic seal, preventing reinfection [1, 2].

Achieving optimal debridement and obturation can be challenging, particularly in teeth with anatomical variations such as developmental grooves, extra roots, or additional canals. Clinicians may overlook these variations, especially when relying solely on conventional two‐dimensional radiography [3]. Previous studies indicate that maxillary central incisors typically present with one single root and canal [4, 5, 6]; however, rare cases with two [7, 8, 9, 10], three [11, 12], four [13, 14, 15], or even five canals [16] have also been reported. Such aberrations are often associated with developmental anomalies, including gemination, fusion, dens invaginatus, supernumerary roots, or cleft lip and palate‐related dental variations [9, 16, 17, 18, 19].

Due to their low prevalence, additional canals in maxillary central incisors may remain undetected without advanced imaging. Cone‐beam computed tomography (CBCT) provides precise three‐dimensional visualization, enhancing the identification of complex root canal anatomical variations that are often missed with conventional radiography [3].

This case report describes endodontic management and aesthetic rehabilitation of a fractured maxillary central incisor with aberrant anatomy, diagnosed using CBCT.

## 2 Case History

**FIGURE 1:**
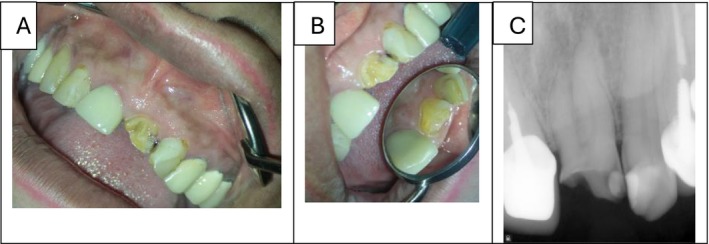
Preoperative clinical and radiographic views of the tooth #21. (A and B) Clinical photograph showing a fractured crown with a carious lesion. (C) Periapical radiograph revealing root bifurcation in the mesiodistal direction.

**FIGURE 2:**
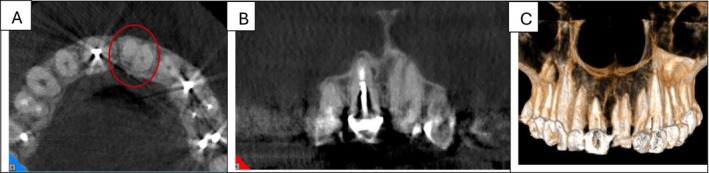
CBCT images confirming the two‐rooted morphology of tooth #21, with two distinct canals and apical foramina. The axial (A) and cross‐sectional (B) CBCT views clearly demonstrate this unusual morphology. (C) Volumetric 3D reconstruction from the CBCT data.

A 73‐year‐old female patient with no significant medical history presented to the Department of Endodontics at the School of Dentistry. Intraoral examination revealed a fractured and carious crown in tooth #21 (Figures 1A & 1B). Application of cold spray (Cold Spray Luber, Iran) on tooth #21 elicited a sharp, severe pain that lingered for over 45 s; while the control teeth responded within the normal limits. The patient reported no pain on palpation and percussion; also, periodontal assessment showed no abnormal probing depth or mobility. A periapical radiograph was obtained, which revealed bifurcated roots in the mesiodistal direction, along with no apical radiolucency or periodontal ligament widening (Figure 1C). To further evaluate the root morphology and confirm the suspected bifurcation, a limited field‐of‐view CBCT scan was performed in accordance with AAE/AAOMR guidelines for the assessment of complex root canal anatomy [20]. The scan confirmed a two‐rooted configuration with two distinct canals and apical foramina (Figure 2). Based on these findings, a diagnosis of symptomatic irreversible pulpitis with normal apical condition was established.

## 3 Treatment

**FIGURE 3:**
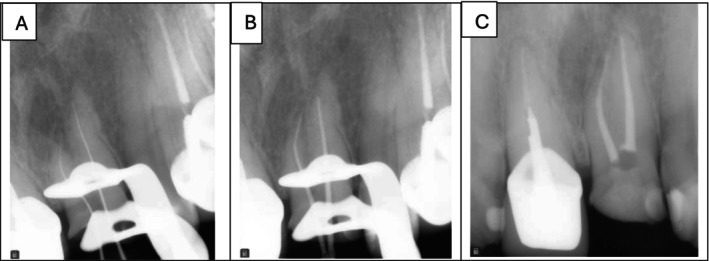
Endodontic procedural radiographs. (A) Working length radiograph. (B) Master cone fit radiograph. (C) Post‐obturation radiograph.

After obtaining written informed consent from the patient, root canal treatment was initiated under local anesthesia (0.9 mL of 2% lidocaine with 1:80,000 epinephrine; Darupakhsh, Tehran, Iran). The tooth was isolated with a rubber dam. An access cavity was prepared under a dental operating microscope magnification (Carl Zeiss, Germany) using a high‐speed long‐shank round diamond bur (No. 1, Jota AG, Rüthi, Switzerland) under continuous water cooling. Ultrasonic tips were employed to refine the access cavity, aiding in localization of the second root canal. A glide path was established with a #15 K‐file (Mani Silk, Mani Inc., Japan), followed by cervical third preparation using a #2 Gates‐Glidden drill (Dentsply Maillefer, Switzerland). The working length was determined electronically (Woodpex III Apex Locator, Woodpecker, China) and confirmed radiographically, measuring 16.5 mm for the mesio‐labial canal and 18 mm for the disto‐labial canal (Figure 3A).

**FIGURE 4:**
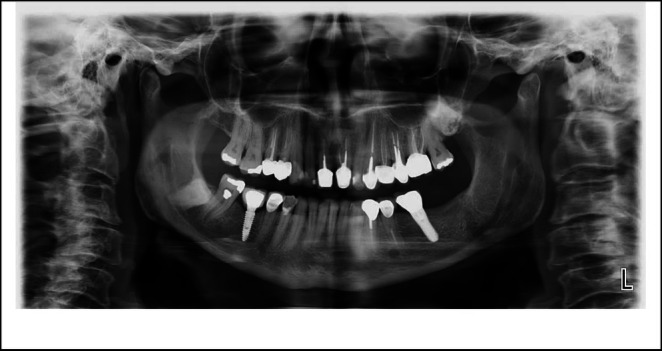
One‐year postoperative panoramic radiograph. The patient was asymptomatic.

The root canals were prepared with ProTaper Gold rotary files (Dentsply Sirona, Ballaigues, Switzerland) using the crown‐down technique. Chemomechanical preparation was performed up to size F2 for both canals. Copious irrigation was performed using alternating 20 mL of 5.25% sodium hypochlorite (per canal) and saline, delivered via a 30‐gauge side‐vented needle. Each NaOCl rinse was followed by 20 s of ultrasonic activation (Woodpecker, China). Final irrigation consisted of 1 mL of 17% EDTA (Merck, Germany) per canal with ultrasonic activation for 20 s, followed by a final saline flush to remove the chelating agent. The master cone fit was radiographically verified before the obturation (Figure 3B). The canals were then filled with gutta‐percha and AH‐26 sealer (Dentsply Sirona, Ballaigues, Switzerland) using the cold lateral compaction technique. Post‐obturation radiographs were obtained to evaluate the quality of the root canal filling (Figure 3C). Temporary restorative material (Cavisol, Tehran, Iran) was applied to establish a temporary coronal seal, and the patient was referred for permanent restoration. The management of this case differed from that of a typical single‐rooted maxillary central incisor due to the presence of two distinct roots concealed beneath a clinically normal crown morphology. Identification of the second canal orifice required careful access cavity refinement under dental operating microscope magnification and the adjunctive use of ultrasonic tips. Separate working lengths and canal trajectories necessitated meticulous negotiation and preparation to avoid procedural errors. These considerations underscore the importance of magnification, enhanced illumination, and conservative dentin removal when treating teeth with atypical internal anatomy. At the 1‐year follow‐up, the tooth was clinically and radiographically asymptomatic (Figure 4).

## 4 Conclusion

Successful endodontic treatment of this two‐rooted maxillary central incisor highlighted the critical role of CBCT in diagnosing complex root canal anatomies, and the effectiveness of modern endodontic techniques in managing such variations. While maxillary central incisors are conventionally single‐rooted, this case emphasized the importance of thorough diagnostic protocols, including advanced imaging, to identify rare anatomical deviations. The use of magnification, precise instrumentation, and tailored obturation technique ensured an optimal outcome. Clinicians should maintain a high index of suspicion for anatomical variations, even in seemingly routine cases, to achieve long‐term therapeutic success and preserve natural dentition. This report adds to the growing body of literature documenting atypical root morphologies and their clinical implications.

## 5 Discussion

Maxillary central incisors are conventionally described as single‐rooted teeth with one single root canal [1]. However, anatomical variations—such as additional roots or canals—although rare, pose significant diagnostic and therapeutic challenges. Such deviations may arise from disturbances in the Hertwig's epithelial root sheath during development [21], and are frequently associated with anomalies like gemination [22], fusion [9], dens invaginatus [15], macrodontia [23], and enamel hypoplasia [24, 25]. Also, this anomaly may result from intrusive luxation of the deciduous predecessor, causing division of the cervical loop and subsequent formation of mesiodistally‐oriented bifid roots in the permanent successor [1]. While most studies confirm that maxillary central incisors are typically single‐rooted [4, 5, 6], a growing body of case reports documents variants with two or more canals [7, 8, 11, 12, 13, 14, 15, 16]. This underscores the necessity for careful preoperative assessment even in seemingly routine cases. Although clinical crown anomalies may signal complex radicular anatomy, this case demonstrates that a typical crown morphology does not preclude multiple roots.

For the assessment of complex root canal anatomy, CBCT remains the diagnostic gold standard. It significantly enhances the detection of morphological variations often not evident on conventional radiographs, thereby enhancing procedural certainty and improving long‐term outcomes. Nevertheless, due to increased radiation exposure, its use should be judicious and guided by clinical indication. In the present case, CBCT was prescribed based on radiographic evidence of root bifurcation, in alignment with AAE/AAOMR guidelines [20] for evaluating suspected unusual root morphology. It played a critical role in confirming the diagnosis and guiding safe, effective treatment. In resource‐limited settings, multiple parallax radiographs (e.g., tube‐shift technique) may serve as a pragmatic, albeit less precise [2, 3].

The successful management of multi‐canaled maxillary central incisors relies on appropriate preparation and obturation methods [1]. In this case, cleaning and shaping were performed using the crown‐down technique to optimize coronal debridement and apical irrigant delivery. Obturation was completed with the cold lateral compaction technique, selected for its clinical predictability. While various alternative techniques, including hybrid thermo‐mechanical, Tagger's, thermoplastic injection, and continuous wave compaction, have been documented for similar anatomies [13, 26, 27, 28, 29, 30, 31], the specific technique is not consistently reported in all published cases [3, 23]. This highlights the value of detailed methodological reporting, as provided here, for clinical guidance.

This case report presented a rare case of a maxillary central incisor with two root canals, unrelated to developmental anomalies such as gemination, fusion, or dens invaginatus, that were ruled out through clinical and radiographic examination. Although most reported cases of multi‐canal maxillary central incisors were associated with anatomical anomalies [9, 15, 22, 32, 33], a few described this variation as an independent morphological trait [7, 10, 13]. These findings reinforce the necessity of thorough diagnostic protocols and adaptive treatment strategies to ensure successful endodontic management.

Also, in this case, we observed that the use of magnification and illumination, along with proper access cavity extension and ultrasonic tips, was proven essential in safely locating the canal orifices, and negotiating, disinfecting, and preparing the canals to their apical foramen.

## Author Contributions

**Mohsen Aminsobhani:** conceptualization, methodology. **Maryam Babaahmadi:** validation, writing – original draft, writing – review and editing.

## Funding

The authors have nothing to report.

## Ethics Statement

For clinical cases, the local ethics committee considers that the patient's consent is sufficient.

## Consent

Written informed consent was obtained from the patient to publish this report in accordance with the journal's patient consent policy.

## Conflicts of Interest

The authors declare no conflicts of interest.

## Data Availability

The data supporting the findings of the present study are available from the corresponding author upon request.
